# Understanding the theoretical and clinical basis of staging models in eating disorders

**DOI:** 10.1192/j.eurpsy.2026.10380

**Published:** 2026-07-31

**Authors:** F. Fernandez-Aranda, S. Jimenez-Murcia

**Affiliations:** Clinical Psychology, University Hospital of Bellvitge-IDIBELL and CIBERobn, Hospitalet del Llobregat, Spain

## Abstract

**Abstract:**

Staging models in psychiatry conceptualize mental disorders as evolving conditions that progress through identifiable phases, each characterized by distinct clinical, cognitive, and biological features. This framework—rooted in developmental psychopathology, clinical course research, and precision‑medicine principles—supports earlier detection, stratified care, and interventions matched to illness stageearly.

In the field of eating disorders, emerging evidence demonstrates that anorexia nervosa, bulimia nervosa, and binge‑eating disorder follow trajectories marked by progressive neuropsychological impairment, metabolic changes, and increasing psychosocial burden. Early stages typically involve modifiable risk factors and mild symptom expression, whereas later stages feature entrenched cognitive rigidity, medical complications, and functional decline and lack of quality of life. Applying staging models enables more accurate prognostic assessment and facilitates stage‑appropriate therapeutic strategies that can reduce chronicity and improve long‑term outcomes.

The presentation will also introduce and discuss the **Catalan Staging Model for Eating Disorders**, a framework developed to integrate clinical staging with a **region‑wide, stepped‑care system**. The model delineates specific stages—from at‑risk presentations to severe, persistent ED—and links each stage to corresponding care pathways, including primary‑care detection, specialized outpatient programs, intensive community treatment, and advanced multidisciplinary care for complex and longstanding cases. This approach exemplifies how staging can be operationalized in real‑world healthcare systems to enhance accessibility, continuity, and precision in ED management.

**Disclosure of Interest:**

None Declared

